# Stakeholders’ Perspectives on the Financial Sustainability of the HIV Response in Nigeria: A Qualitative Study

**DOI:** 10.9745/GHSP-D-22-00430

**Published:** 2023-04-28

**Authors:** Daniel Ogbuabor, Caroline Olwande, Iris Semini, Obinna Onwujekwe, Yewande Olaifa, Chioma Ukanwa

**Affiliations:** a Department of Health Administration and Management, Faculty of Health Sciences and Technology, University of Nigeria, Enugu Campus, Enugu, Nigeria.; b Joint United Nations Programme on HIV/AIDS, Abuja, Nigeria.; c Equitable Financing Practice, Joint United Nations Programme on HIV/AIDS, Geneva, Switzerland.; d National Agency for the Control of AIDS, Abuja, Nigeria.; e National AIDS, Sexually Transmitted Infections, and Hepatitis Control Programme, Federal Ministry of Health, Abuja, Nigeria.

## Abstract

**Background:**

Countries in sub-Saharan Africa, including Nigeria, continue to depend on donor funding to achieve their national HIV response goals. The Government of Nigeria has made limited progress in translating political commitment to reduce donor dependency into increased domestic investment to ensure the sustainable impact of the HIV response. We explored the context-specific factors affecting the financial sustainability of the HIV response in Nigeria.

**Methods:**

Between November 2021 and March 2022, we conducted document reviews (n=13) and semistructured interviews with purposively selected national and subnational stakeholders (n=35). Data were analyzed thematically using the framework of health financing functions comprising revenue generation, pooling, and purchasing.

**Results:**

Stakeholders reported that there is a low level of government funding for the HIV response, which has been compounded by the weak engagement of Ministry of Finance officials and the unpredictable and untimely release of budgeted funds. Opportunities for domestic funding include philanthropy and an HIV Trust Fund led by the private sector. Integration of HIV treatment services into social health insurance schemes has been slow. Commodity purchasing has been inefficient due to ineffective coordination. Government stakeholders have been reluctant to support one-stop-shop facilities that target key and priority populations.

**Conclusion:**

Opportunities exist in the government and private sectors for improving domestic health financing to support transitioning from donor support and ensuring the financial sustainability of the HIV response in Nigeria. To ensure that domestic financing for the HIV response is stable and predictable, the amount of domestic funding needs to increase and a framework that incorporates donor transition milestones must be developed, implemented, and monitored.

## Abstract (precis)

The authors’ explored stakeholders’ perspectives on factors that affect the financial sustainability of the HIV response in Nigeria when donors withdraw. The findings can help decision-makers to develop, implement, and evaluate HIV financial sustainability plans.

## Teaser

**Key Findings:**

- The financial sustainability of the HIV response in Nigeria faces a variety of challenges, including heavy reliance on donor funding that is declining, a low level of domestic funding, and a low level of private-sector financing.
- In an effort to improve financing sustainability, integrating HIV testing and treatment services into social health insurance schemes has been slow due to concerns over the high cost of antiretroviral drugs.
- Purchasing of HIV treatment and prevention commodities has been inefficient because of the lack of an integrated procurement and supply chain management process.
- Despite the high costs associated with providing HIV treatment in hospitals, decentralizing treatment to primary health care centers and shifting tasks to less specialized staff has been slow.

**Key Implications:**

- Decision-makers and donors can use these findings to develop and implement a plan to increase domestic resource mobilization.
- The findings can help guide the implementation of a country-owned sustainable HIV response framework that incorporates donor transition milestones, ensures evidence-informed integration of HIV services into universal coverage schemes, initiates policy change to enable public financing of the community-led response, prioritizes HIV care and treatment, and addresses inequalities in future resource allocation decisions.

## INTRODUCTION

The financial sustainability of the HIV response in sub-Saharan Africa (SSA) is a growing concern because of the transitions in demographics, diseases, donor development assistance, and domestic health financing.^1^ The demographic transition, characterized by an aging population with increased demands coexisting with a growing adolescent population, entails that people living with HIV (PLHIV) stay longer on treatment, while new infections might be increasing among adolescents (in the absence of a vaccine), as well as marginalized and vulnerable populations. Disease transition represents the shift in the burden of disease from infectious diseases to noncommunicable diseases and injuries, a shift that results in increasing multimorbidity among PLHIV and poses a huge burden on the health care system.^1^ Donor development transition signifies the countries that are shifting away from relying on external aid as their national gross domestic product per capita improves, despite their weak domestic capacity to handle post-aid shocks.^1^ Domestic financing transition refers to the countries that are raising domestic resources to finance their health system as they transition out of external aid, especially by prioritizing health in government budgets and increasing public health care spending channeled through prepaid pooled mechanisms.^1^ Financial instability, exacerbated by the COVID-19 pandemic, combined with the war in Ukraine and the debt crisis, has further constrained the fiscal space in more than 60 countries’ ability to invest in health.^2^

Despite an increase in domestic financing of the HIV response during the last decade, SSA countries continue to depend on external aid, and a large gap exists between available financing and the funding needed to achieve global HIV goals.^3^ To continue progress toward addressing HIV as a public health threat and sustain the progress of domestic and donor investments, all African governments, regardless of their ability to pay, must take measures to establish pathways and actions toward a successful transition from development assistance to domestic financing of the HIV response.^4^^,^^5^ Yet, the Government of Nigeria has made limited progress in translating political commitment to reduce donor dependency into increased domestic resources to ensure the sustainable impact of the HIV response.

Despite increased domestic financing for the HIV response in SSA, countries continue to depend on external aid.

Sustainability means that a health program or country can maintain and scale up service coverage to a level that will provide continuing control of the health problem even after the withdrawal of external funding.^6^ In the context of HIV, sustainability implies that governments can lead and manage the response by developing a country-led sustainability roadmap, supporting country visions, closing the funding gap to achieve the goal of eliminating HIV as a public health threat and maintaining impact, effectively using local capacity, improving alignment across donors and governments, finding inefficiencies, addressing inequalities, and integrating into and contributing to resilient health systems.^7^ In other words, when countries move away from donor-dependent financing, they can successfully expand their national HIV response, reach epidemic control, and maintain the control after the transition while having robust systems and decision-making processes in place to ensure high and equitable coverage of target populations.^8^

Evidence of the effects of donor transitions on the HIV response has shown mixed results. In India, after the removal of external aid, the government sustained service coverage and support for key and priority populations.^9^ Nevertheless, the shortfalls of the national health system, including limited flexibility in program management, delays in funding, commodity stock-outs, and community member perceptions of a narrowing program focus, reflected the health financing and public financial management challenges occasioned by the lack of national preparedness when the donors transitioned.^9^ Similarly, in SSA, donor transition resulted in low quality of care,^4^^,^^5^^,^^10^^–^^13^ decreased access to preventive and community outreach services,^10^^,^^12^^–^^14^ reduced retention in care,^5^^,^^10^^,^^11^^,^^13^ decreased access to laboratory services,^4^^,^^10^ staff shortages and loss of financial incentives for health workers,^4^^,^^5^^,^^10^ and reliance on user fees for previously free HIV services.^5^^,^^10^ The negative effects of transitioning from a donor-funded HIV response point to a need for early sustainability planning that examines the integration of the HIV response in the health care system and identifies policy, programmatic, and financing measures to mitigate systemic bottlenecks and to maintain the impact and quality of an effective and equitable HIV response.

The pathway toward a sustainable HIV response requires using a complex and multipronged approach that considers financial, epidemiological, political, structural, and programmatic factors, as well as human rights. However, this study focuses on financial sustainability, described as the availability of stable and diverse funding mechanisms to finance the HIV response.^13^ In other words, revenue generation must be predictable, have effective and efficient pooling mechanisms, and include strategic purchasing of HIV goods and services.^15^ Current guidance and assessments that have examined different HIV response sustainability plans and transition readiness have yielded insufficient country-specific geographic, population, and programmatic guidance on how to plan for the financial sustainability of donor-sponsored HIV programs after donors withdraw.^16^

Previous studies have highlighted several factors that affect domestic resource mobilization during and after donor transitions. In Western Cape, South Africa, the lack of guidance and communication around the pace of the donor’s budget decrease constrained the government’s ability to finance the HIV program budget after the donor transition.^17^ While multiple funding streams enhanced the sustainability of HIV treatment programs, unpredictable domestic public financing reduced its sustainability.^17^^,^^18^ Private philanthropic aid contributed to the Ugandan HIV response, whereas earmarked taxes were also important sources of additional funding for the HIV response in several African countries.^19^^,^^20^ Some countries have created dedicated HIV Trust Funds—an approach that has recently been used in Nigeria. However, in Uganda, this approach contributed insignificantly to the financial sustainability of the HIV response because the tax revenue earmarked to capitalize the Trust Fund covered just 0.5% of the annual HIV resource needs.^18^ An HIV Trust Fund that was led by the private sector seemed more successful than those that depended on government allocations.^18^

Many countries have integrated HIV services into health care financing systems and universal coverage schemes to improve the financing of their HIV response.^20^^–^^23^ In Uganda, employer-provided medical insurance and special HIV insurance schemes have been used to provide service coverage to PLHIV.^20^ In Thailand and Vietnam, social health insurance (SHI) schemes have covered HIV prevention, care, and treatment services to improve equity in health care access and financing.^23^ In India, 2 types of SHI schemes exist.^22^ The first type is a community-led, HIV-specific scheme covering only PLHIV, with a small pool of beneficiaries, little or no risk pooling, limited financial protection, high premium-low coverage mix, and low sustainability. The second type is an HIV-sensitive scheme, which is subsidized by the government, offers a wider scope of coverage across populations and across disease conditions, attracts public subsidies, and has a high potential for cost recovery and sustainability.^22^

In Uganda, purchasing of HIV services and commodities was enhanced by integrating external grants into national budgeting for medicines financing^19^ and geographic prioritization policy, a priority-setting process in which public budgets reflected the national burden of disease.^5^ In Uganda and Bangladesh, voucher schemes improved access to HIV testing for key and priority populations (KPs), including men who have sex with men and vulnerable and poor populations.^20^^,^^24^ A differentiated service delivery model improved the efficiency of providing HIV treatment services in sub-Saharan Africa.^25^^,^^26^ In Vietnam, purchasing SHI-funded HIV treatment services did not result in financial hardship for PLHIV because few PLHIV incurred out-of-pocket costs, representing less than 5% of the total national health expenditure.^23^ However, the effective and efficient use of SHI schemes to purchase HIV care and treatment services in Vietnam was hampered by supply-side challenges, including health workers’ poor attitudes, poor quality of care and treatment services, and a lack of guidance on SHI coverage of care and treatment for PLHIV.^27^

The lack of methods to assess and address challenges for integrating the HIV response into health care budgets and financing mechanisms is a critical shortfall that this study aims to address. For example, despite the high SHI coverage of PLHIV in Vietnam, the lack of information about SHI schemes, high premium affordability, and difficult enrollment procedures limited the coverage of PLHIV by the SHI scheme.^27^^,^^28^ Similarly, most research on HIV donor transition in Africa focuses on the effect of loss of donor support in South and East African countries.

The lack of methods to assess and address challenges for integrating the HIV response into health care budgets and financing mechanisms is a critical shortfall that this study aims to address.

In Nigeria, published studies on transitioning away from donor financing have focused on its effects on HIV services,^10^ differentiated care,^26^ sustainability of the supply chain system,^29^ and transition readiness.^16^ Other Nigerian studies have shown that high HIV prevalence has reduced life expectancy,^30^ diminished real gross domestic product growth rate,^31^ decreased productivity,^32^^,^^33^ and been associated with low government spending.^34^ While the context and the broader ecological system within which health care systems operate matter,^35^ studies investigating the contextual factors that might shape financial sustainability in Nigeria are scarce. Therefore, this study explored stakeholders’ perspectives on contextual factors that affect the financial sustainability of the HIV response in Nigeria when donors withdraw. This information can be used by planners and decision-makers to develop, implement, and evaluate HIV financial sustainability plans in Nigeria.

## METHODS

### Conceptual Framework

The study explores the HIV response integration into Nigeria’s health care financing guided by the World Health Organization framework of health financing functions comprising revenue generation, pooling and fund management, and purchasing, together with benefit specification and governance arrangements.^15^ Revenue generation encompasses the different sources of HIV funds, including government budgets, households (out-of-pocket spending), the private sector, and external aid. The level of integration depends on what share of the HIV response funds draws from the same revenue source as other health area funds (e.g., TB response or immunization). Pooling and fund management involves how prepaid funds are redistributed and coordinated to support purchasing of HIV services, including whether the HIV funds are pooled and managed with other health funds. The degree of purchasing integration is based on whether the transfer of HIV and other health funds from purchasers to providers relied on the same channels and mechanisms. The study takes the perspective of strategic purchasing. Appropriate governance arrangements are prerequisites for equitable resource distribution, policies, efficiency, transparency, accountability, and ultimately, the sustainability of the HIV response that ensures adequate funding allocations and utilization relative to needs, financial protection, quality, and equity.

### Study Setting

We conducted this study in Nigeria, a lower-middle-income West African country with a population of more than 200 million people. Nigeria comprises 36 states and a Federal Capital Territory. Nigeria has 1.74 million PLHIV comprising 92% adults (55% females and 37% males) and 8% children. The 2020 estimates show that 90% of PLHIV aged 15–64 years know their HIV status, 86% of PLHIV who know their status are on antiretroviral therapy (ART), and 72% of PLHIV on ART are virally suppressed. However, the proportion of pregnant women who accessed services to prevent mother-to-child transmission services declined from 50% in 2019 to 44% in 2020.^36^^,^^37^

Nigeria’s HIV response is coordinated by the National Agency for the Control of AIDS (NACA). Within the Federal Ministry of Health, the National HIV and STI Control Programme is responsible for the health sector response. At the subnational level, there are 37 State Agencies for the Control of HIV/AIDS and State HIV and STI Control Programmes. In terms of financing, the National Health Insurance Authority (NHIA) exists alongside autonomous SHI schemes in 36 states and the Federal Capital Territory. Nigeria spent 3.2% of its gross domestic product on health in 2019.^39^ HIV expenditure was 0.12% of the gross domestic product in 2021.^38^ The government expenditure on health was 4.1% of overall government expenditure in 2019.^38^ Domestic government HIV expenditures was 0.16% of total government expenditure in 2021.^38^ In 2020, 67% of HIV expenditures was donor funded.^38^ The HIV response will cost about US$1.2 billion, US$1.5 billion, and US$2.2 billion, respectively, from 2022 to 2027, at baseline (5%), moderate (50%), and aggressive (90%) scale-up.^39^ The cost of inaction is estimated at 0.19%, 0.15%, and 0.12% of the gross domestic product, respectively, at baseline (5%), moderate (50%), and aggressive (90%) scale-up of interventions.^39^

### Research Design

This study adopted a qualitative, exploratory design to complement efforts toward the financial sustainability of the HIV response in Nigeria.^40^ Successful transitions from donor to domestic funding require focused engagement on the part of all stakeholders, and qualitative methods are critical to capturing the complex interactions in sustainability drivers and related policy decisions.^41^ Additional data were collected through a targeted desk review.

### Study Population and Sampling Strategy

The study population comprised stakeholders involved in HIV/AIDS financing in Nigeria purposively selected to maximize the diversity of implementation experiences and to strengthen understanding of how contextual and institutional factors influenced the financing of the HIV response. We selected stakeholders at the national level (e.g., NACA, National HIV and STI Control Programme, NHIA, and Federal Ministry of Health) (n=8) and state level (State Agencies for the Control of HIV/AIDS, SHI schemes, and State HIV and STI Control Programmes) (n=18), as well as representatives of international partners (n=5), private-sector organizations (n=2), and civil societies (n=2). The stakeholders were mapped in consultation with officials of NACA and the National HIV and STI Control Programme. Overall, 35 participants were interviewed based on their positions, involvement in HIV response, health financing experience, and willingness to participate in the study.

### Data Collection Methods

We developed a semistructured, in-depth interview guide based on the conceptual framework of this study to conduct interviews. The interview guide explored the challenges of implementable strategies for increasing the domestic resources for the HIV response, pooling and management of funds, purchasing, and public financial management, as well as the political economy surrounding the HIV response in Nigeria. We conducted the interviews between November 2021 and March 2022 (Table 1). Appointments were sought by cell phone or personal visits. We interviewed stakeholders in their offices (n=18) and over the phone (n=17). We were unable to secure interviews with certain high-level government officials due to scheduling conflicts. Although the quality of phone interviews might seem lower than the quality of in-person interviews,^42^ this was not the case because we used an experienced interviewer. Interviews, which lasted about 45–60 minutes, were conducted in English and audio-recorded. Data collection was stopped when additional interviews revealed no new insights.

**TABLE 1.** National and Subnational Stakeholders Interviewed About the HIV Response in Nigeria

**Stakeholder Category**	**Participants, No.**
Federal level	8
Federal Ministry of Health	
National Agency for the Control of AIDS	
National HIV and STI Control Programme	
National Health Insurance Authority	
Federal Ministry of Finance	
State Agencies for the Control of AIDS (Akwa Ibom, Benue, Enugu, Gombe, Kaduna, and Lagos states)	6
State HIV/AIDS and STIs Control Programmes (Akwa Ibom, Enugu, Gombe, Kano, Kogi, and Taraba states)	6
State Health Insurance Schemes (Benue, Delta, Kano, Niger, Oyo, and Yobe states)	6
International partners	5
World Health Organization	
Joint United Nations Programme on HIV/AIDS	
United States Agency for International Development	
Health Policy Plus	
Civil societies	2
Health Reform Foundation of Nigeria	
Network of Persons Living with HIV/AIDS in Nigeria	
Private sector	2
Nigeria Business Coalition Against AIDS	
Development Governance International Consult	
Total	35

Additional data were collected through document review. In consultation with key government officials and development partners, we identified 13 policy and program documents that were relevant to the research questions.

### Data Analysis

Data were analyzed using a framework approach that involved coding, mapping, and organizing the data under common themes and interpretation.^43^ NVivo 11 software was used for coding and categorizing the data. Codes were developed from the conceptual framework and reading of the data. Coding was done by 2 persons guided by a coding framework, which enhanced the validity and reliability of this study.^44^ All differences in coding were reconciled by consensus. The findings from different sources were compared for patterns of convergence and divergence. Furthermore, the study findings were discussed with study participants and key health systems stakeholders in a stakeholders’ meeting.

### Ethical Approval

The Health Research Ethics Committee of Enugu State Ministry of Health, Enugu, Nigeria, approved this study. Participants gave their written informed consent for in-person interviews and verbal informed consent for phone interviews. Participants were assured of the confidentiality of the information they provided during the interviews. The transcripts were anonymized by removing all personal identifiers and replacing them with pseudonyms.

## RESULTS

Table 2 summarizes the themes and subthemes that emerged from the findings across the 3 financing functions: revenue generation, pooling and fund management, and purchasing.

**TABLE 2.** Summary of Emergent Themes and Subthemes From the Findings Across 3 Financing Functions in Nigeria

**Health Financing Functions**	**Themes**	**Subthemes**
Revenue generation	Budget	Low budget size
		Unpredictable release of approved funds
		Nonimplementation of earmarked funds by states
		Lack of geographic prioritization
		Weak engagement with Ministry of Finance officials
	Donor funding	HIV response is donor driven
		Donor funding is declining
	Private-sector financing	Existence of HIV Trust Fund could increase private-sector investment
	Philanthropic contribution	Zakat seen as opportunity to fund vulnerable PLHIV
		Philanthropic aid/adoption of PLHIV enrolled in SHI schemes
Pooling and fund management	Donor coordination	Lack of harmonization, weak partner coordination, and lack of a clear transition plan
	SHI scheme	Existence of national guidelines
		Slow integration of HIV into SHI schemes
		Fear of cost of HIV treatment
	Basic Health Care Provision Fund	HIV services not provided as specified in benefits
		Benefit specification excluded HIV agencies and programs
Purchasing	Community-led interventions	Key populations targeted using one-stop shops
		Unwillingness to sustain one-stop shops
		Reluctance to financing community-led interventions from government budgets
	Efficiency of purchasing	Slow decentralization of HIV treatment to primary health facilities
		Private providers enrolled in SHI schemes to provide HIV services
		Government spending delinked from results

Abbreviations: PLHIV, people living with HIV; SHI, social health insurance.

### Revenue Generation

#### Budget

**FIGURE 1:**
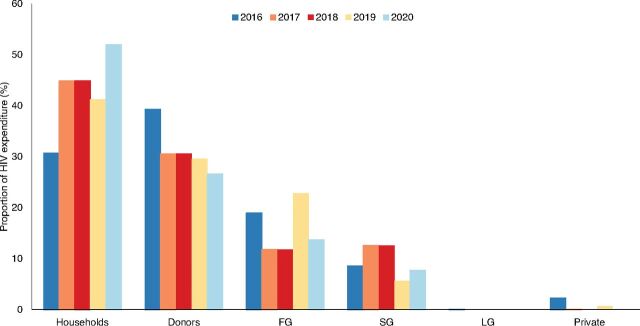
Trend of HIV Expenditure in Nigeria^a^ Abbreviations: FG, federal government; LG, local government; SG, state government. ^a^Data source: National Health Account Reports.

**FIGURE 2:**
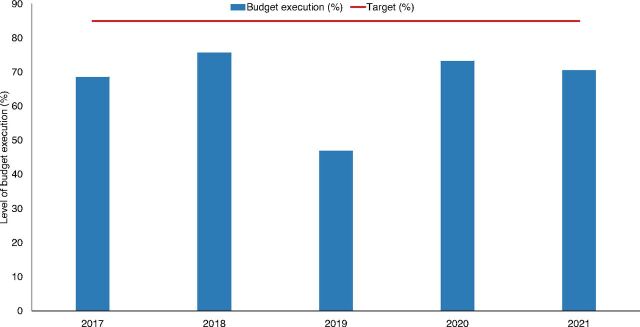
Trend of Budget Execution by the National Agency for the Control of AIDS^a^ ^a^Data source: National Agency for the Control of AIDS report.

Document review showed that public HIV expenditure at all levels of government fluctuated between 2016 and 2021 (Figure 1). State and local government funding was particularly low. Between 2016 and 2021, about 67% (range, 47%–76%) of the Government of Nigeria’s allocation to NACA was spent (Figure 2), below the 85% target set by the National HIV Strategic Framework. The view that public spending on the HIV response was low at federal, state, and local levels of government was consistent across the various groups of stakeholders. HIV budget execution was also perceived to be low and inefficient due to low political commitment. In instances when the budget was increased, most participants noted that the release of the funds was unpredictable. Unspent funds were commonly returned to the treasury. The document review indicated that 0.5%–1.0% of federal allocation to states was earmarked for the state-level HIV response. However, participants explained that implementation of the earmarked fund was stalled because of a change in the government in 2019 and because the funds were not deducted at the source.

*Although the National Economic Council approved the appropriation of 0.5%–1% states’ federal monthly allocation to HIV response in 2017, only about 4 states have been able to implement this resolution.* —Policymaker, national level

Both government and nongovernment stakeholders noted that government budgets neither matched the needed services nor reflected the burden of disease. Document review indicated that government budgets prioritized geopolitical representation instead of geographical areas with high HIV burden. Participants identified three drivers of poor priority-setting. First, the budgets were historical. Secondly, the budgets were not program based. Further, the Ministry of Health did not sufficiently engage the Ministry of Finance. The participants also highlighted the need for political advocacy at the federal and state levels to improve priority-setting.

Both government and nongovernment stakeholders noted that government budgets neither matched the needed services nor reflected the burden of disease.

*There is a gap between the people who understand the epidemiology of the response and those who appropriate the budget for the HIV response.* —International partner

*The engagement between the health and finance/budget ministries, departments, and agencies has been weak.* —Policymaker, Ministry of Finance

#### Donor Funding

**FIGURE 3:**
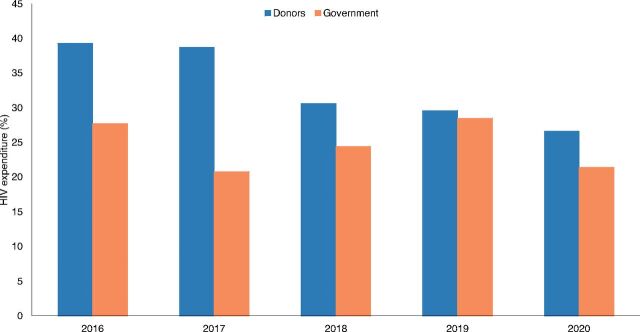
Trend of Donor and Government HIV Expenditure in Nigeria

**FIGURE 4:**
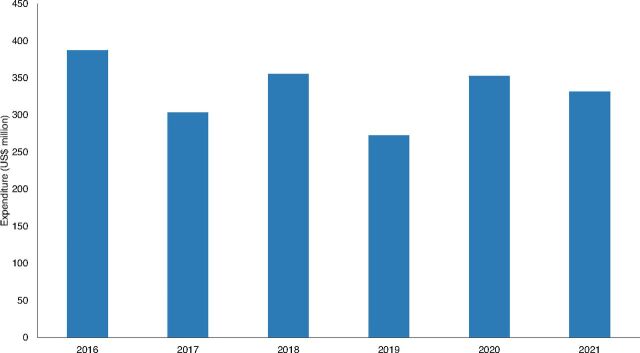
Trend of PEPFAR’s HIV Expenditure in Nigeria^a^ Abbreviation: PEPFAR, U.S. President’s Emergency Program for AIDS Relief. ^a^Data source: PEPFAR Panorama Spotlight.

The U.S. President’s Emergency Program for AIDS Relief and the Global Fund are the leading financiers of Nigeria’s HIV response. A review of the National Health Account Reports indicates that external funding decreased from about 39% of the total HIV expenditure in 2016 to almost 27% in 2020 (Figure 1). Comparatively, domestic public funding marginally increased from 21% in 2017 to 29% in 2019 but declined to about 21% in 2020 (Figure 3). The funding from the U.S. President’s Emergency Program for AIDS Relief declined from US$387 million (2016) to US$331 million in 2021 (Figure 4). All categories of stakeholders stated that HIV response is donor driven. Nonetheless, donor funding was reported to be declining due to donor fatigue and donor transition.

*The national AIDS spending assessment from 2014 to 2018 show that external donors cover most of the HIV expenditure.* —Policymaker, national level

#### Private-Sector Financing

Document review indicated that private-sector financing for HIV is low, ad hoc, and tokenistic. Most stakeholders were optimistic that the HIV Trust Fund, a newly launched private-sector–led funding mechanism, would considerably increase private-sector investment in the HIV response. Document review highlighted that the HIV Trust Fund was geared to initially fund the scaling up of services to prevent mother-to-child transmission of HIV with plans for future expansion to fund procurement of HIV commodities and pediatric HIV treatment. Nongovernment stakeholders argued for an accountability framework that should be integrated into government funding management to support the implementation of the trust fund.

*There must be strong accountability framework, transparency, and where everybody knows how much is generated, and how the resources are utilized.* —Stakeholder, private sector

#### Philanthropic Contribution

Document review indicated that philanthropists could contribute about US$2 million annually to the HIV response from 2021 to 2025. Some stakeholders perceived that altruistic payment of SHI contributions by philanthropic individuals could improve funding of the HIV response. The stakeholders suggested that individual philanthropists can “adopt” PLHIV in their communities and remit their premiums directly to SHI schemes. Similarly, a state-level policymaker suggested that Zakat, a religious obligatory donation by Muslims to the poor and needy, amounting to about 2.5% of an eligible Muslim’s wealth, could be used to help fund the HIV response for orphans and vulnerable children. However, participants noted that because these contributions were voluntary, they would likely be unpredictable.

### Pooling and Fund Management

#### Donor Coordination

Most government stakeholders observed a lack of harmonization and weak coordination among international development partners. Document review indicated that donors managed their funds based on their work plan, were not accountable to the government, and did not have a transition plan.

Most government actors observed a lack of harmonization and weak coordination among international development partners.

*The Planning Commission at the national and state levels must enforce the country’s compact strategy and hold donors accountable in line with Paris declaration and Accra agenda for action.* —Policymaker, national level

Conversely, an international partner stakeholder offered a different perspective.

*The current alignment between the Global Fund and PEPFAR [U.S. President’s Emergency Program for AIDS Relief], the largest funders for HIV, has cut costs, reduced duplication, and improved efficiency of purchasing of HIV commodities.* —International partner

#### SHI Scheme

Most stakeholders reported that SHI schemes do not provide comprehensive HIV services at the national and state levels. Document review revealed that a national strategy for integrating HIV services into SHI schemes exists with various options and a minimum service package, but implementation has been slow due to the high cost of HIV treatment, lack of leadership by NHIA, and lack of funds.

*The fear is that the cost of drugs will deplete the pool of resources.* —Policymaker, national level

*The state cannot cover HIV services when the national health insurance scheme has not.* —Policymaker, state level

*The premium has remained static despite increased minimum wage and retirement without recruitment which means that state schemes lack the fund to expand coverage.* —Policymaker, state level

#### Basic Health Care Provision Fund

Document review showed that the Basic Health Care Provision Fund benefits consist of 9 interventions, including HIV testing and counseling for pregnant women, ART for mothers and newborns, and infant feeding counseling. A national benefit package integrating HIV services into the Basic Health Care Provision Fund has been developed and adopted. However, HIV agencies and departments at the national and state levels were not involved in developing the benefits.

*What is written on paper has not been translated into practice.* —Policymaker, national level

### Purchasing

#### Community-Led Interventions

Most nongovernment stakeholders highlighted the usefulness of one-stop shops in reaching KPs (i.e., female sex workers, men who have sex with men, and people who inject drugs and their partners) with HIV services. Document review indicated that a one-stop shop is a facility that provides HIV prevention, care, treatment, and support services in a safe and nonjudgmental environment in which KPs feel free to access HIV services. Estimates of KPs guide the location, number, and distribution of one-stop shops. But due to outdated existing data on KPs and lack of a sufficient budget for new KP surveys, one-stop shops poorly target KPs.

*The estimates of KPs keep changing in terms of size and location because of demographic shifts in hot spots.* —International partner

Alternatively, government stakeholders preferred the establishment of KP-friendly health facilities. They perceived one-stop shops to be both stigmatizing and at possible risk of regulatory challenges from health facility regulatory bodies.

Nongovernment stakeholders advocated for the government to fund civil society organizations to implement community-led interventions using government budgets.

*Civil societies should serve as implementing partners for HIV activities, such as one-stop shops, covered in government budgets.* —International partner

However, government stakeholders argued that funding civil society organizations would limit their autonomy.

*There must be a clear procedure to ensure that mutual accountability and conflict of interests are clearly displayed by both parties.* —Policymaker, national level

#### Efficiency of Purchasing

Most stakeholders stated that decentralizing HIV treatment to primary health care centers (PHCs) has been slow despite the high cost associated with providing ART in hospitals. Document review pointed out that promoting access to ART in health centers and health posts and payment using capitation could reduce the cost of HIV treatment. Stakeholders were optimistic about using PHCs to increase access to and improve coverage of HIV services, as well as reduce the cost of care, but were concerned about shifting tasks traditionally performed by doctors to less specialized staff. Document review indicated that a patient-centered approach of differentiated care where stable PLHIV received multi-month medication from the PHCs closest to them resulted in cost savings for providers and patients alike. Most participants opined that the Basic Health Care Provision Fund was an opportunity to increase access to HIV services in PHCs. Nevertheless, most stakeholders perceived coordination to be weak among the multiple agencies involved in decentralizing HIV services to PHCs at the national and state levels.

Stakeholders were optimistic about using PHCs to increase access to and improve coverage of HIV services, as well as reduce the cost of care.

*PHCs can increase access to testing and treatment using the Basic Health Care Provision Fund model of 1 testing center per ward.* —Policymaker, national level

All categories of stakeholders agreed on the need to expand the involvement of the private sector in the HIV response. Document review indicated that the bulk of HIV funding was spent on externally sourced antiretroviral drugs, yet a business case for local manufacturing of HIV commodities was lacking. Furthermore, stakeholders stated that private providers who are enrolled in the SHI schemes could expand HIV testing and treatment services.

*NACA can leverage the NHIA medicine supply initiative to mobilize private-sector resources for local manufacture of HIV commodities and negotiate down the price.* —Policymaker, national level

Most stakeholders agreed that when government and donors align investments in the HIV response, costs could be saved and duplications reduced. Eliminating “middlemen” and dealing directly with the manufacturers was perceived by stakeholders to achieve efficiency gains. Nongovernment stakeholders revealed that the government spent heavily on travel and training that do not bear directly on the program outcomes (e.g., prevalence and incidence), leaving gaps in service delivery, supply chain, test kits, antiretroviral drugs, surveillance activities, and laboratory services. Stakeholders attributed the persistence of health facility user fees for HIV services to a lack of HIV commodities and supplies.

*Spending on traveling, workshops, and training are important but should not take as much as 70%–80% of programmatic cost.* —International partner

*The truth is that there are some out-of-pocket payments being done in the facilities.* —Policymaker, state level

## DISCUSSION

This study aimed to investigate the contextual factors that affect the financial sustainability of the HIV response in Nigeria from the perspectives of national and subnational stakeholders. Several key findings on revenue generation, pooling and fund management, and purchasing of HIV services warrant further exploration.

Our finding that domestic public spending on HIV response is low is consistent with the low domestic public financing for HIV and health in SSA.^4^^,^^17^^–^^20^ This finding implies that budget allocation to HIV will remain low unless there is a change in political commitment to implement domestic financing strategies that increase public spending on the HIV response; integrate HIV prevention, care, and treatment into universal health coverage initiatives; and guarantee financial risk protection for all PLHIV.^20^ The Government of Nigeria must increase its budget size and improve budget execution to sustain the HIV response needed as donor funds decline despite the government’s tight macrofiscal context. To achieve these 2 budget outcomes, officials of HIV departments and agencies must communicate the previous budget performance to budget officials at the Ministry of Finance. The budget must also shift from geopolitical representation to geographic prioritization, reflecting the burden of disease. The shift from geographic representation to geographic prioritization itself carries political connotations, with power dynamics between states and the federal government against a data-based analysis of where the burden is highest. At the national level, the Federal Ministry of Health must ensure that the HIV resource needs estimate is used to inform the government’s fiscal policy and expenditure framework. The programmatic and financial resources of federal and state governments need to align to finance the HIV response. Engagement with Nigeria’s Governors Forum will facilitate the appropriation of earmarked funds for the HIV response at the state and local levels. Further, the release of approved funds must be predictable to ensure timely access to funds. Finally, a sustainable framework that incorporates donor transition milestones must be developed, implemented, and monitored.

Engagement with Nigeria’s Governors Forum will facilitate the appropriation of earmarked funds for the HIV response at the state and local levels.

Consistent with evidence from other countries,^13^ Nigeria launched the HIV Trust Fund as an additional financing mechanism targeting services for the prevention of mother-to-child transmission of HIV. The stakeholders’ concern that private-sector financing is not sustainable is supported by evidence from Uganda that the AIDS trust fund contributed insignificantly to the financial sustainability of the HIV response.^18^ Nevertheless, Nigeria’s private-sector–led trust fund might succeed in raising considerable funds to finance the HIV response because HIV Trust Funds that have depended on private endowments seem to have been more successful than those that have depended on government allocations.^18^ This may be more likely, given the recent experience with a robust private-sector endowment fund for the COVID-19 response in Nigeria.^45^

The finding that philanthropic aid might contribute to financial sustainability is comparable to the contribution of private philanthropic aid to the Ugandan HIV response.^19^^,^^20^ However, the private philanthropic donations in the current study were domestic, unlike the Ugandan experiences that were mostly from nonbilateral, external sources. Our finding is also supported by evidence of willingness to pay for antiretroviral drugs for other household members.^46^ In Nigeria, individuals can support the HIV response in diverse ways, including through the SHI adoption model to pay for coverage of orphans and vulnerable children and poor populations.^47^ The model is designed to target a pool of public-spirited individuals with a high net worth to pay premiums for low-income and vulnerable citizens, thereby increasing their access to prepaid health care.^47^ A second option is to channel Zakat, a religious contribution, to the HIV Trust fund. In addition, individuals can donate money for treatment adherence and nutrition support for PLHIV. Nonetheless, the sustainability of domestic private donations is constrained by its lack of predictability in the amount of funding and lack of stability in terms of funding flow emanating from distrust for government programs.^47^^,^^48^ Therefore, sustained advocacy and sensitization are required to improve the buy-in of philanthropists.

Our findings that SHI schemes in Nigeria do not provide comprehensive HIV services suggest a need to integrate HIV services into the benefits package of the schemes. These findings contrast with evidence of integrating HIV services into the national universal coverage schemes in South Africa and Thailand, special HIV insurance in Uganda, and HIV-sensitive schemes in India.^20^^–^^22^ Capitalizing on the NHIA’s renewed mandate, state SHI schemes and the Basic Health Care Provision Fund provide opportunities for integrating HIV into universal coverage schemes in Nigeria. A standard package of HIV services for all publicly funded schemes should be guided by the national blueprint for the integration of HIV services into SHI schemes and should involve all related stakeholders. The premium for the SHI schemes should be actuarially determined and periodically reviewed to reflect salary adjustments.

A standard package of HIV services for all publicly funded schemes should be guided by the national blueprint for the integration of HIV services into SHI schemes and should involve all related stakeholders.

Despite the alignment of donor and government procurement of HIV commodities, we found that Nigeria has no country-owned sustainable HIV response framework that incorporates donor transition milestones. Our finding is comparable to evidence from South Africa.^17^ A lack of a time-bound transition plan seems to have provided the Government of Nigeria with contradictory incentives to invest public funds into the HIV response. This is contrary to the Nigerian experience with the immunization program where Gavi, the Vaccine Alliance and the Government of Nigeria agreed to a transition plan.^49^ Consistent with evidence from other countries,^50^ low political will to reprogram donor-funded services to domestic sources, weakness in estimating HIV response resource needs, and stigma against KPs combined with a low political will to fund community-led interventions underlie the lack of a transition plan in Nigeria. Consequently, a transition plan is needed that incorporates the donor’s budget decrease to guide the development and implementation of a financial sustainability plan for Nigeria’s HIV response. To facilitate the development and implementation of a transition plan in Nigeria, a robust coordination mechanism is needed that provides an opportunity for stakeholder engagement, high-level advocacy informed by resource needs estimates and fiscal space analysis, and political commitment to community-led interventions.^49^

The finding that government stakeholders were opposed to one-stop-shop facilities to target KPs suggests that these populations may have less access to services when Nigeria transitions from donor support. This finding is compounded by the reluctance of government officials to finance community-led interventions with government budgets. Our finding is consistent with decreased access to preventive and community outreach services found in other studies in Nigeria and Uganda.^10^^,^^12^^–^^14^ In contrast, continued government support for KPs was observed in the Indian HIV response after the donor transition.^9^ The Government of Nigeria endorsed the 2021 political declaration on HIV and has domesticated the 2021–2026 Global AIDS Strategy into its National HIV Strategic Framework and Plan. The Government of Nigeria committed to achieve the 2025 global targets of 30-60-80 for community-led responses for HIV testing and treatment, HIV prevention, and program support achievement of social enablers. Given that one-stop shops are community-led interventions for KPs who contribute to about 40% of new HIV infections in Nigeria,^36^ the government should consider sustaining one-stop shops and increasing the proportion of HIV services delivered by communities.

The findings highlighted a slow decentralization of HIV treatment to PHCs. Our finding contrasts with the evidence that shifting tasks from doctors to less specialized staff lowered the costs of HIV treatment with comparable outcomes.^51^^,^^52^ Also, shifting responsibility from doctors to adequately trained and supported nurses or community health workers for managing HIV patients does not decrease the quality of care.^53^^,^^54^ Given the efficiency gains in employing nurses or community health workers instead of doctors, decision-makers should strengthen the task-shifting policy to accelerate the decentralization of HIV treatment to PHCs.

Furthermore, our findings show the low involvement of private providers in the delivery of HIV care and treatment. Existing evidence suggests that although private providers have less disruption of services after donor transition, reduced HIV clinical care in private facilities increases the burden on public facilities.^55^ In Nigeria, private providers are already involved in the delivery of HIV treatment. Also, some private providers are enrolled in NHIA and SHI schemes.^56^^,^^57^ Engaging and incentivizing private-sector facilities might improve strategic purchasing of HIV services, especially for those who already use private health facilities.

Engaging and incentivizing private-sector facilities might improve strategic purchasing of HIV services, especially for those who already use private health facilities.

The findings identified purchasing of HIV services and commodities to be inefficient due to duplications, ineffective coordination, and weak priority setting. In Nigeria, the alignment of donor and government procurement of HIV commodities has begun, but a single integrated procurement and supply chain management process has not been established. Besides, public spending on human resources and other recurrent activities is high and not commensurate with the service delivery outcomes. Lowering the unit costs of HIV commodities and human resources might result in budgetary savings that could be reinvested in the HIV response.^58^ Therefore, planners and decision-makers must prioritize allocative efficiency in HIV care and treatment in future resource allocation decisions.

### Strengths and Limitations

This study adds stakeholders’ views of policies that are intended to improve the financial sustainability of donor-supported programs in Nigeria to the growing scholarship on donor transitions in SSA. Reflecting the views of stakeholders at different levels—national and subnational levels as well as government and nongovernment sectors—is a key strength of this study. Secondly, this study uses the framework of health financing to broaden the perspective on financing strategies, including revenue generation, pooling, benefit specification, purchasing, and governance imperatives for transitioning from donor support. Nonetheless, we may have excluded some stakeholders, especially health facility–based service providers. Future studies should investigate providers’ perspectives on the effect of transitioning from donor support.

## CONCLUSION

Increasing the “pie piece” of HIV response financing is warranted. Opportunities exist in the government and nongovernment sectors for improving domestic health financing to support the financial sustainability of the HIV response in Nigeria. Our findings indicate a need for increased budget size and improved budget execution, an effectively managed and accountable private-sector–led trust fund, and an increase in philanthropic investment to ensure stable and predictable domestic financing for the HIV response. Additionally, pooling and fund management must prioritize the integration of HIV services into universal coverage schemes and partner coordination. Further, strategic purchasing must promote efficiency, financial protection, community-led interventions, engagement of primary health care and private providers, and result-driven resource allocation. Finally, a sustainable framework that incorporates donor transition milestones must be developed, implemented, and monitored.

## Notes

**Peer Reviewed**

**First published online:** March 29, 2023.
